# Nonmotor Symptoms Differ Between Essential Tremor and Tremor‐Dominant Parkinson's Disease

**DOI:** 10.1002/brb3.70288

**Published:** 2025-02-09

**Authors:** Mingqiang Li, Runcheng He, Xun Zhou, Yuzheng Wang, Qiying Sun, Chunyu Wang, Sheng Zeng, Lifang Lei, Heng Wu, Shanqing Yi, Jun Wen, Qian Xu, Jifeng Guo, Beisha Tang, the China Essential Tremor Alliance

**Affiliations:** ^1^ Department of Neurology, Multi‐Omics Research Center for Brain Disorders The First Affiliated Hospital, Hengyang Medical School University of South China Hengyang Hunan China; ^2^ The First Affiliated Hospital, Clinical Research Center for Immune‐Related Encephalopathy of Hunan Province, Hengyang Medical School University of South China Hengyang Hunan China; ^3^ Department of Neurology Xiangya Hospital, Central South University Changsha Hunan China; ^4^ Department of Geriatric Neurology Xiangya Hospital, Central South University Changsha Hunan China; ^5^ National Clinical Research Center for Geriatric Disorders, Xiangya Hospital Central South University Changsha Hunan China; ^6^ Department of Neurology, The Second Xiangya Hospital Central South University Changsha Hunan China; ^7^ Department of Geriatric Neurology, The Second Xiangya Hospital Central South University Changsha Hunan China; ^8^ Department of Neurology, The Third Xiangya Hospital Central South University Changsha Hunan China; ^9^ Department of Neurology The First People's Hospital of Changde Changde Hunan China

**Keywords:** essential tremor, hyposmia, nonmotor symptom, tremor‐dominant Parkinson's disease

## Abstract

**Background:**

Distinguishing between essential tremor (ET) and tremor‐dominant Parkinson's disease (PD‐TD) can be challenging due to overlapping motor symptoms. This study aims to investigate the differences in nonmotor symptoms (NMS) between ET and PD‐TD patients to provide additional evidence for differentiating these two conditions.

**Methods:**

This retrospective study included 1656 participants, comprising 558 PD‐TD patients, 584 ET patients, and 514 controls. ET patients were assessed using the Tremor Research Group Essential Tremor Rating Assessment Scale (TETRAS), while PD‐TD patients were evaluated based on the Unified Parkinson's Disease Rating Scale (UPDRS). All participants were assessed for NMS using the Nonmotor Symptoms Scale (NMSS).

**Results:**

The composite NMSS score for the PD‐TD group was significantly higher than that for the ET group and the control group (23.44 ± 20.20 vs. 12.60 ± 14.89 vs. 9.37 ± 12.44, *p* < 0.001). Compared to ET patients, PD‐TD patients had an increased risk of all NMS, especially in hyposmia (OR = 7.70, 95% CI: 5.11–11.62). The NMSS score, urinary symptoms, and hyposmia may play a role in differentiating ET from PD‐TD. The area under the curve (AUC) is 0.766 (95% CI: 0.739–0.793), with a sensitivity of 80.8% and specificity of 58.6%. When family history is included in the analysis, the AUC increases to 0.819 (95% CI: 0.795–0.843), with sensitivity improving to 82.4% and specificity to 68.2%.

**Conclusions:**

The study reveals significant differences in NMS between ET and PD‐TD. Compared to patients with ET, those with PD‐TD exhibit more frequent and severe NMS. NMS and family history are helpful in differentiating between ET and PD‐TD.

## Graphical abstract

This study highlights significantly higher nonmotor symptoms (NMS) in tremor‐dominant Parkinson's disease (PD‐TD) compared to essential tremor (ET), particularly hyposmia (OR = 7.70, 95% CI: 5.11–11.62). NMSS scores, urinary symptoms, and hyposmia may help distinguish PD‐TD from ET. The area under the curve (AUC) is 0.766 (95% CI: 0.739–0.793), with 80.8% sensitivity and 58.6% specificity. Including family history improves the AUC to 0.819 (95% CI: 0.795–0.843), with sensitivity at 82.4% and specificity at 68.2%.

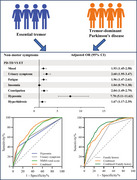

## 1 Introduction

Essential tremor (ET) and Parkinson's disease (PD) are common movement disorders whose prevalence increases with age, affecting approximately 4.6% and 1.8% of the population over 65 years old, respectively (de Rijk et al. 2000; Louis 2023; Pringsheim et al. 2014; Welton et al. 2021). ET is primarily characterized by action tremor of the upper limbs (Shanker 2019), while resting tremor is more common in PD patients (Balestrino and Schapira 2020). However, clinical observations have identified a degree of symptom overlap between ET and PD. For example, between 88% and 92% of PD patients experience action tremor (Mailankody, Netravathi, and Pal 2017; Thenganatt and Louis 2012), and 20%–30% of ET patients may present with resting tremor (Thenganatt and Louis 2012). Some patients with ET may also exhibit “soft neurological signs” such as questionable dystonic posturing (Bhatia et al. 2018; Louis et al. 2020), and there is a potential for ET to progress into PD (Tarakad and Jankovic 2018). These factors complicate the accurate diagnosis of these two diseases. The tremor‐dominant PD (PD‐TD), characterized predominantly by tremor (Thenganatt and Jankovic 2014), further blurs the clinical distinction between PD and ET, with up to 37%–50% of tremor cases being misdiagnosed at initial presentation (Jain, Lo, and Louis 2006).

Several studies have explored different approaches to distinguish between ET and PD. In addition to the evaluation of clinical symptoms, various experimental techniques have been applied, such as neurophysiology, neuroimaging, and fluid biomarker assessments (Angelini, Paparella, and Bologna 2024; Arabia et al. 2018; Heim et al. 2022; Y. Huang et al. 2022; Lin et al. 2023; Purrer et al. 2023; Wang et al. 2023; Yoon et al. 2016). These methods show potential in aiding differential diagnosis, particularly in the early or prodromal stages of the diseases. Furthermore, olfactory testing has also been investigated as a valuable supplementary tool for differentiating ET from PD (Elhassanien et al. 2021). The differentiation between ET and PD primarily relies on the evaluation of clinical symptoms, particularly the differences in motor symptoms. In fact, aside from motor symptoms, patients with ET and PD experience various nonmotor symptoms (NMS), such as mild cognitive impairment, depression, anxiety, and sleep disorders, which often appear in the early stages of the disease (Louis 2016; Titova and Chaudhuri 2018). Identifying these distinct NMS may aid in differentiating ET from PD‐TD. Research indicates that ET and PD‐TD may exhibit distinct NMS profiles (Kwon et al. 2016; Shalash et al. 2021), yet due to the limited number of studies and small sample sizes of included patients, the precise differences in NMS between them remain contentious. Given this background, this study is dedicated to exploring the differences in NMS between ET and PD‐TD, aiming to provide additional criteria for distinguishing between the two.

## 2 Methods

### 2.1 Patients and Study Design

This retrospective study, conducted from May 2020 to May 2023, involved the continuous evaluation of participants recruited through outpatient and inpatient departments across 19 clinical centers in China. The study excluded patients diagnosed with secondary Parkinsonism, acquired or physiological tremors, dystonia, and other types of tremor disorders. The control group was composed of healthy volunteers, ensuring through evaluation that they did not have any significant neurological diseases, nor did they or their family members have a history of ET, PD, or other tremor conditions. All participants underwent comprehensive neurological examinations and assessments. The clinical demographic data collected included gender, age, age at onset (AAO) of disease, disease duration, medical history, family history (ET probands have blood relatives with ET, and PD probands have blood relatives with PD), and both motor and nonmotor symptoms, which were obtained from the China Parkinson's Disease and Movement Disorder Multi‐Center Database and Collaborative Network (PD‐MDCNC, http://pd‐mdcnc.com) (Sun et al. 2023; X. Zhou et al. 2022). This study received approval from the Medical Ethics Committee of Xiangya Hospital, Central South University, and was conducted in strict accordance with the ethical principles of the Declaration of Helsinki. Informed consent was obtained from all participants.

### 2.2 ET Criteria

The diagnostic process for individuals with ET is guided by the criteria put forth by the International Parkinson and Movement Disorder Society (MDS) (Bhatia et al. 2018). In evaluating tremor severity, the assessment employs the Tremor Research Group Essential Tremor Rating Assessment Scale (TETRAS) (Elble et al. 2012). This scale is bifurcated into two segments: TETRAS Part I, which quantifies the tremor's impact on the functionality of daily living, and TETRAS Part II, which delineates the tremor by type (inclusive of postural and kinetic tremors), its spatial distribution (spanning head, facial regions, vocal cords, limbs, and trunk), and its intensity. The grading on the TETRAS scale is directly proportional to the severity of tremor manifestations, with higher scores indicating greater severity.

### 2.3 PD Criteria

The diagnosis of PD patients adhered to the MDS Clinical Diagnostic Criteria (Postuma et al. 2015), utilizing the Unified Parkinson's Disease Rating Scale (UPDRS) and its associated subscores, as well as the Hoehn and Yahr Scale (H&Y) for staging the disease. The tremor score (calculated as the sum of items 20 and 21 from Part III of the UPDRS divided by 4) and the akinetic/rigid score (the sum of items 22–27 and item 31 from Part III of the UPDRS divided by 15) ratio was computed. When this ratio is greater than or equal to 1, patients are classified as having the PD‐TD (Kang et al. 2005). The study included PD‐TD patients with Hoehn and Yahr Scale ≤ 2.5.

### 2.4 Scale Assessment

Within the framework of this investigation, a meticulous process of medical history documentation and neurological evaluations was carried out for all subjects, with the Nonmotor Symptoms Scale (NMSS) serving as the primary tool for quantifying NMS (Chaudhuri and Martinez‐Martin 2008). The NMSS, encompassing a diverse set of 30 questions, spans nine pivotal domains: cardiovascular, sleep/fatigue, mood/cognition, perceptual problems/hallucination, memory/attention, gastrointestinal tract, urinary, sexual, and miscellaneous symptoms. The frequency of each NMS was then calculated based on the proportion of patients who scored ≥ 1 on each item or domain of the NMSS. The Mini‐Mental State Examination (MMSE) was utilized to assess cognitive function (Folstein, Folstein, and McHugh 1975).

### 2.5 Statistical Analysis

This study's statistical analyses were meticulously executed using SPSS software, version 26.0. Prior to the analytical phase, an elaborate process was undertaken to code, cleanse, and verify the integrity of the dataset. For the presentation of data, continuous variables were expressed as mean ± standard deviation, and categorical variables were delineated in percentages. The analytical strategy incorporated the use of chi‐square tests for categorical variables and Mann–Whitney *U* tests for continuous variables to facilitate comparative analysis between groups. Further comparative evaluations among the ET, PD‐TD, and the control cohorts on continuous variables were conducted employing generalized linear models (GLM), with adjustments for pertinent confounders such as age, gender, body mass index (BMI), and AAO. Using both univariate and multivariate logistic regression analyses, we identified risk factors for NMS in ET and PD‐TD to construct a clinical diagnostic prediction model. The diagnostic performance of the model was quantified using the area under the receiver operating characteristic (ROC) curve (AUC). Statistical significance was determined at a threshold of *p* < 0.05.

## 3 Results

### 3.1 Clinical Characteristics of the Study Population

This retrospective investigation encompassed 1656 Chinese adults, including 558 PD‐TD patients, 584 ET patients, and 514 individuals in the control group (Table 1). Gender distribution across the PD‐TD and ET cohorts did not reveal statistically significant differences, though a slight male predominance was noted in the PD‐TD group. The groups presented distinct average ages: 60.35 ± 9.83 years for PD‐TD, 53.80 ± 16.21 years for ET, and 60.93 ± 19.79 years for the control group, underscoring significant age disparities. ET patients demonstrated an earlier AAO compared to PD‐TD counterparts (42.17 ± 17.00 vs. 56.53 ± 10.11, *p* < 0.001), with ET also exhibiting a prolonged disease course (11.63 ± 10.25 years vs. 3.82 ± 3.64 years for PD‐TD, *p* < 0.001). A notable difference was the higher incidence of familial history among ET patients compared to those with PD‐TD (48.63% vs. 10.93%, *p* < 0.001).

**TABLE 1:** Demographic and clinical characteristics of the study population.

Items	Control (514)	PD‐TD (558)	ET (584)
Male, *n* (%)	310 (60.31)	313 (56.09)	295 (50.51)^*^
Age (years)	60.93 ± 19.79	60.35 ± 9.83^*^	53.80 ± 16.21^*^, ^**^
Disease duration (years)	—	3.82 ± 3.64	11.63 ± 10.25^**^
Age at onset (years)	—	56.53 ± 10.11	42.17 ± 17.00^**^
Family history (%)	—	61 (10.93)	284 (48.63)^**^
BMI (kg/m^2^)	23.05 ± 3.27	23.03 ± 3.17	23.05 ± 3.08
Smoking (%)	167 (32.50)	154 (27.60)	137 (23.46)^*^
Alcohol consumption (%)	103 (20.04)	121 (21.68)	112 (19.18)
Hypertension (%)	170 (33.07)	148 (26.52)^*^	130 (22.26)^*^
Diabetes mellitus (%)	50 (9.73)	41 (7.34)	63 (10.79)^**^
Hyperlipidemia (%)	54 (10.51)	53 (9.50)	59 (10.10)
Asymmetry of tremor (%)	—	401 (71.86)	160 (23.97)^**^
Head (%)	—	10 (1.79)	191 (32.71)^**^
Face (%)	—	141 (25.27)	162 (27.74)
Upper limbs (%)	—	542 (97.13)	584 (100.00)^**^
Resting tremor (%)	—	463 (82.97)	87 (14.90)^**^
Postural/kinetic tremor (%)	—	360 (64.52)	584 (100.00)^**^
Lower limbs (%)	—	396 (70.97)	154 (26.37)^**^
Tremor severity			
TETRAS‐I	—	—	14.58 ± 9.86
TETRAS‐II	—	—	19.29 ± 8.88
H&Y off	—	1.76 ± 0.54	—
UPDRS			
UPDRS I	—	1.75 ± 1.56	—
UPDRS II	—	9.01 ± 4.21	—
UPDRS III	—	23.45 ± 12.00	—

*Note*: Data for continuous variables are presented as mean ± standard deviation.

Abbreviations: BMI, body mass index; ET, essential tremor; H&Y scale, Hoehn and Yahr scale; PD‐TD, tremor‐dominant Parkinson's disease; TETRAS, Tremor Research Group Essential Tremor Rating Assessment Scale; UPDRS, the Unified Parkinson's Disease Rating Scale.

* *p* < 0.05 compared to control.

** *p* < 0.05 compared to PD‐TD.

Moreover, limb tremor asymmetry was markedly more pronounced in PD‐TD patients than in ET patients (71.86% vs. 23.97%, *p* < 0.001). When examining tremor distribution, ET patients more frequently reported head tremor than their PD‐TD counterparts, although facial tremor incidence remained comparable between groups. PD‐TD patients, however, experienced a greater prevalence of lower limb tremors. Distinctions in tremor types were evident, with PD‐TD largely manifesting as resting tremors, whereas ET was predominantly associated with postural/action tremors, illustrating the nuanced clinical presentations within these cohorts.

### 3.2 Comparison of NMS Severity Among PD‐TD, ET, and Controls

Table 2 delineates the profile of NMS among the cohorts. The aggregated analysis underscores a progressive elevation in NMS total scores from the control group through the ET cohort to the PD‐TD group (9.37 ± 12.44 vs. 12.60 ± 14.89 vs. 23.44 ± 20.20), with these differences achieving statistical significance. The PD‐TD group's NMS severity markedly surpasses that observed in the ET and control groups, with the ET group similarly exhibiting enhanced severity over the control, albeit to a lesser extent. Excluding the cardiovascular segment, a comparative analysis across all domains of the NMSS revealed significant disparities among the groups.

**TABLE 2:** NMS characteristics of PD‐TD, ET, and controls.

NMS	Control (514)	PD‐TD (558)	ET (584)	*p* value	Adjusted *p* value (ET vs. Control)^a^	Adjusted *p* value (PD‐TD vs. Control)^a^	Adjusted *p* value (ET vs. PD‐TD)^b^
Cardiovascular	0.43 ± 1.06	0.53 ± 1.36	0.46 ± 1.51	< 0.157	0.167	0.137	0.956
Sleep/fatigue	2.91 ± 4.35	6.09 ± 6.37	3.82 ± 5.15	< 0.001	< 0.001	< 0.001	< 0.001
Mood/cognition	0.67 ± 3.22	3.59 ± 6.37	2.16 ± 4.57	< 0.001	< 0.001	< 0.001	< 0.001
Perceptual problems	0.17 ± 0.70	0.42 ± 1.25	0.11 ± 0.51	< 0.001	0.389	< 0.001	< 0.001
Attention/memory	1.57 ± 3.06	2.58 ± 3.40	2.30 ± 3.36	< 0.001	< 0.001	< 0.001	0.406
Gastrointestinal symptoms	0.78 ± 2.09	2.49 ± 3.57	0.91 ± 2.10	< 0.001	0.04	< 0.001	< 0.001
Urinary symptoms	2.25 ± 4.17	3.60 ± 4.86	1.59 ± 3.42	< 0.001	0.330	< 0.001	< 0.001
Sexual function	0.07 ± 0.78	0.29 ± 1.51	0.13 ± 1.12	0.001	0.190	0.003	0.124
Other symptoms	0.53 ± 1.52	3.84 ± 4.69	1.13 ± 2.64	< 0.001	< 0.001	< 0.001	< 0.001
NMSS total score	9.37 ± 12.44	23.44 ± 20.20	12.60 ± 14.89	< 0.001	< 0.001	< 0.001	< 0.001
MMSE	—	26.87 ± 3.31	27.42 ± 3.05	< 0.001	—	—	0.299

*Note*: Data for continuous variables are presented as mean ± standard deviation.

Abbreviations: ET, essential tremor; NMSS, Nonmotor Symptoms Scale; PD‐TD, tremor‐dominant Parkinson's disease.

^a^ Adjusted age, sex, BMI.

^b^ Adjusted age, sex, BMI, AAO.

**FIGURE 1:**
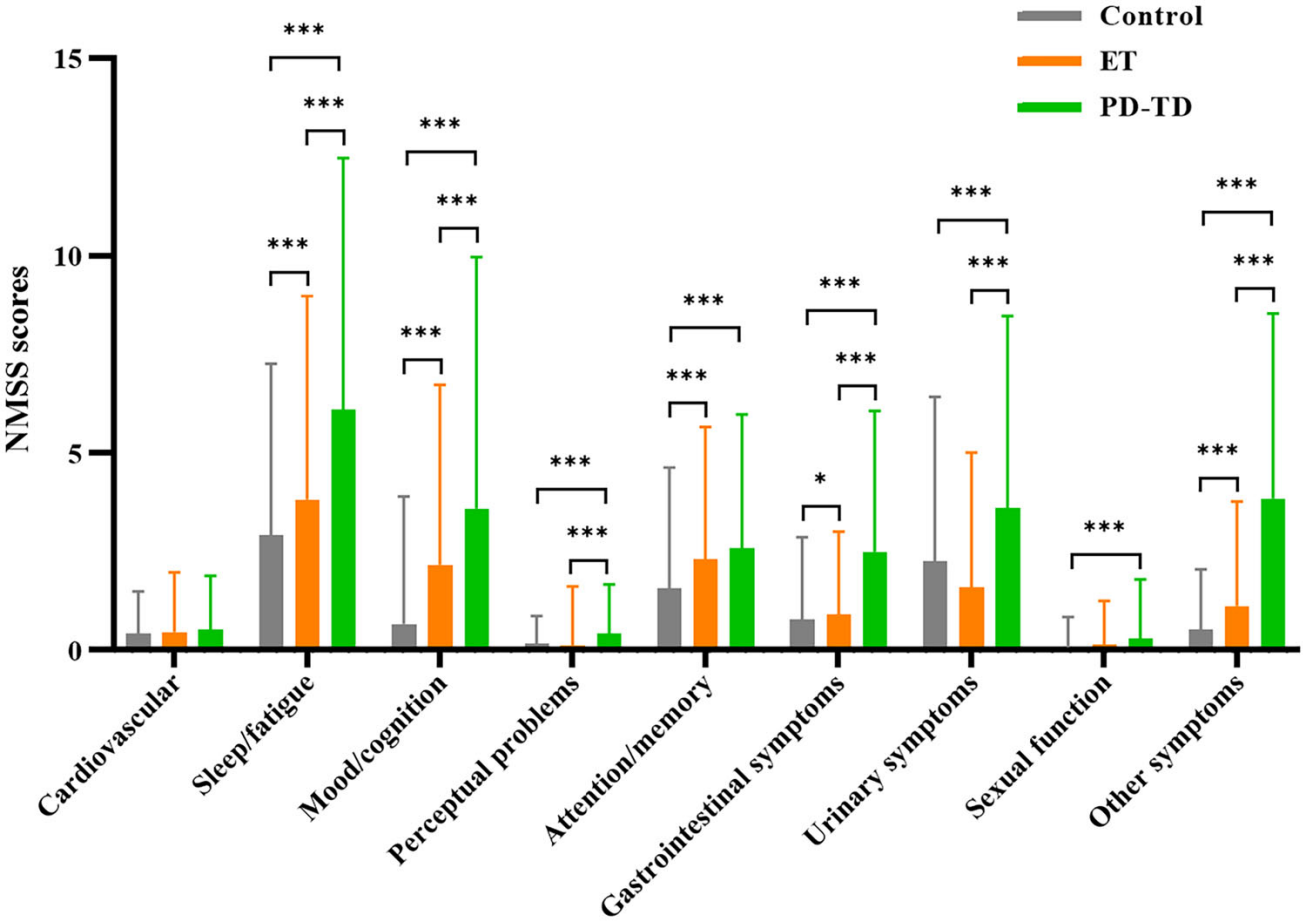
Comparison of NMS severity among PD‐TD, ET, and controls. The comparisons of NMS between ET, PD‐TD, and the control group were adjusted for age, sex, and BMI. For comparisons of NMS between ET and PD‐TD, additional adjustments were made for age at onset.

Subsequent adjusted scoring for NMS (Figure 1) identified no notable discrepancies between ET and control groups within the cardiovascular, perceptual problems, urinary symptoms, and sexual function domains. Conversely, substantial variations were apparent in sleep/fatigue, mood/cognition, attention/memory, gastrointestinal symptoms, and other symptoms' domains. A juxtaposition of PD‐TD and control groups disclosed a singular domain without significant variance (cardiovascular), contrary to all other NMS domains, which diverged significantly. Comparing ET to PD‐TD groups, cardiovascular, attention/memory, and sexual function domains displayed no significant scoring differences, whereas pronounced discrepancies were evident across the remaining domains. The MMSE scores for patients with ET were higher than those for PD‐TD (*p* < 0.001). However, after adjusting for confounding factors, including age, sex, BMI, and AAO, the difference was not statistically significant.

### 3.3 Comparison of NMS Frequency Among PD‐TD, ET, and Controls

Based on the NMSS, we identified seven common NMS as shown in Table 3. Among patients with ET, the most frequent NMS included insomnia (41.95%), mood disturbances (34.76%), urinary symptoms (32.88%), and fatigue (30.99%), with hyposmia being less common (9.24%). In contrast, patients with the PD‐TD more commonly experienced urinary symptoms (60.75%), fatigue (49.10%), and mood disturbances (47.49%). The most frequent NMS in the control group were urinary symptoms (32.88%) and insomnia (22.76%).

**TABLE 3:** Comparison of frequency of NMS between different groups.

				ET vs. Control		PD‐TD vs. Control		PD‐TD vs. ET	
NMS	Control (514)	PD‐TD (558)	ET (584)	OR (95% CI)	Adjusted OR (95% CI)^a^	OR (95% CI)	Adjusted OR (95% CI)^a^	OR (95% CI)	Adjusted OR (95% CI)^b^
Mood	47 (9.14)	265 (47.49)	203 (34.76)	5.24 (3.75–7.48)	5.20 (3.65–7.41)	8.99 (6.38–12.66)	9.18 (6.49–13.01)	1.70 (1.34–2.15)	1.93 (1.45–2.58)
Urinary symptoms	169 (32.88)	339 (60.75)	192 (32.88)	1.00 (0.78–1.29)	1.57 (1.18–2.11)	1.62 (1.27–2.07)	1.87 (1.43–2.44)	3.16 (2.48–4.03)	2.60 (1.95–3.47)
Fatigue	62 (12.06)	274 (49.10)	181 (30.99)	3.27 (2.38–4.50)	4.10 (2.90–5.80)	7.03 (5.14–9.62)	7.69 (5.55–10.66)	2.15 (1.69–2.74)	1.96 (1.47–2.61)
Insomnia	117 (22.76)	246 (44.09)	245 (41.95)	2.45 (1.18–3.19)	3.53 (2.60–4.78)	2.68 (2.05–3.49)	3.40 (2.52–4.58)	1.09 (0.86–1.38)	1.04 (0.79–1.38)
Constipation	46 (8.95)	208 (37.28)	120 (20.55)	2.63 (1.83–3.78)	3.40 (2.29–5.05)	6.05 (4.27–8.56)	8.97 (6.01–13.39)	2.30 (1.77–2.99)	2.04 (1.49–2.79)
Hyposmia	30 (5.83)	223 (39.96)	54 (9.24)	1.64 (1.03–2.61)	2.36 (1.42–3.90)	10.74 (7.16–16.12)	13.80 (8.90–21.39)	6.53 (4.71–9.06)	7.70 (5.11–11.62)
Hyperhidrosis	27 (5.25)	125 (22.40)	96 (16.44)	3.55 (2.27–5.54)	3.70 (2.34–5.85)	5.21 (3.37–8.05)	5.73 (3.65–8.99)	1.47 (1.09–1.97)	1.67 (1.17–2.39)

Abbreviations: ET, essential tremor; PD‐TD, tremor‐dominant Parkinson's disease.

^a^ Adjusted age, sex, BMI.

^b^ Adjusted age, sex, BMI, AAO.

**FIGURE 2:**
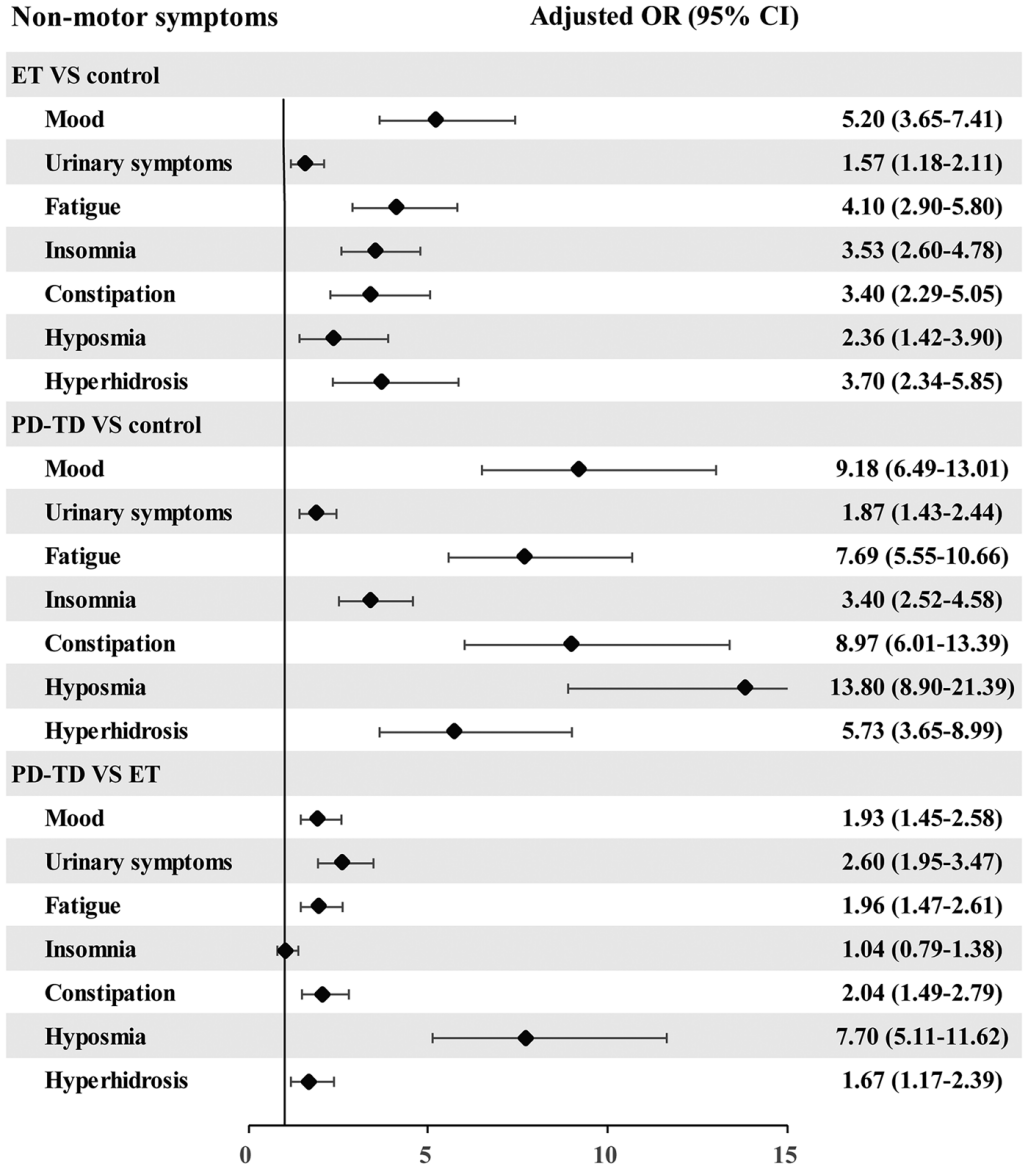
Comparison of NMS frequency among PD‐TD, ET, and controls.

Compared to the control group, the difference in urinary symptoms among ET patients was not significant (OR = 1.00, 95% CI: 0.78–1.29); however, adjusted data indicated a relatively higher risk of urinary symptoms in ET patients (OR = 1.57, 95% CI: 1.18–2.11; Figure 2). ET patients generally had a higher risk of other NMS compared to the control group, especially in terms of mood disturbances (OR = 5.20, 95% CI: 3.65–7.41). PD‐TD patients also exhibited a universally higher risk across all NMS compared to the control group, often exceeding threefold, particularly in the area of hyposmia (OR = 13.80, 95% CI: 8.90–21.39).

Compared to ET patients, PD‐TD patients showed an increased risk in all NMS except for insomnia, with the most significant increase observed in hyposmia, which was 7.7 times higher than in ET patients (OR = 7.70, 95% CI: 5.11–11.62; Figure 2). For other NMS, the risk increase in PD‐TD compared to ET ranged from one to two times, indicating that the most significant difference between PD‐TD and ET patients lies in hyposmia.

**FIGURE 3:**
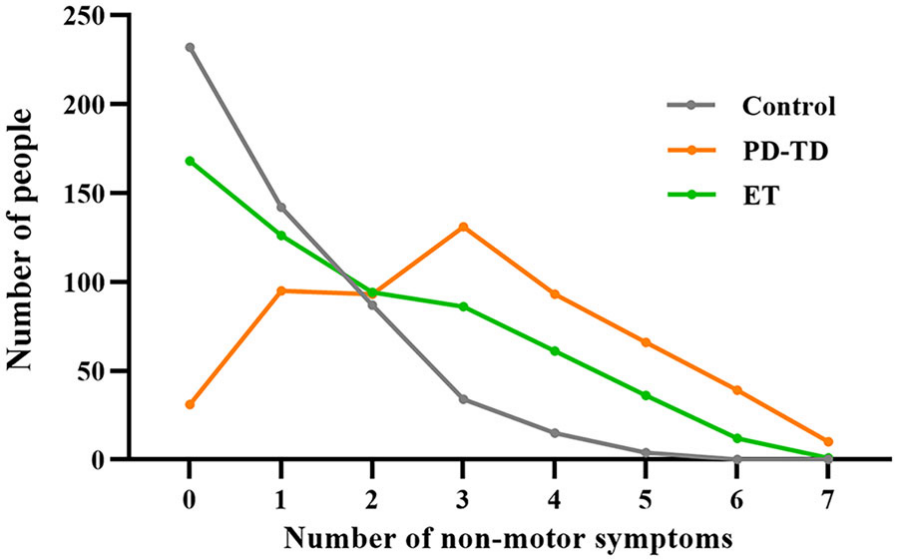
Total number of NMS between ET, PD‐TD and controls.

Table S1 outlines the co‐occurrence of multiple NMS among PD‐TD patients, ET patients, and the control group. Up to 94.44% of PD‐TD individuals reported experiencing at least one NMS, compared to 71.23% of ET patients and 54.86% of control group members. As depicted in Figure 3, PD‐TD patients commonly exhibited between one and four concurrent NMS, with three NMS being the most common scenario. Conversely, both the ET group and the control group showed a decreasing trend in the number of concurrent NMS, with a more pronounced reduction in the control group, where 72.76% of members had no or only one NMS.

### 3.4 Clinical Prediction Models for ET and PD‐TD

**FIGURE 4:**
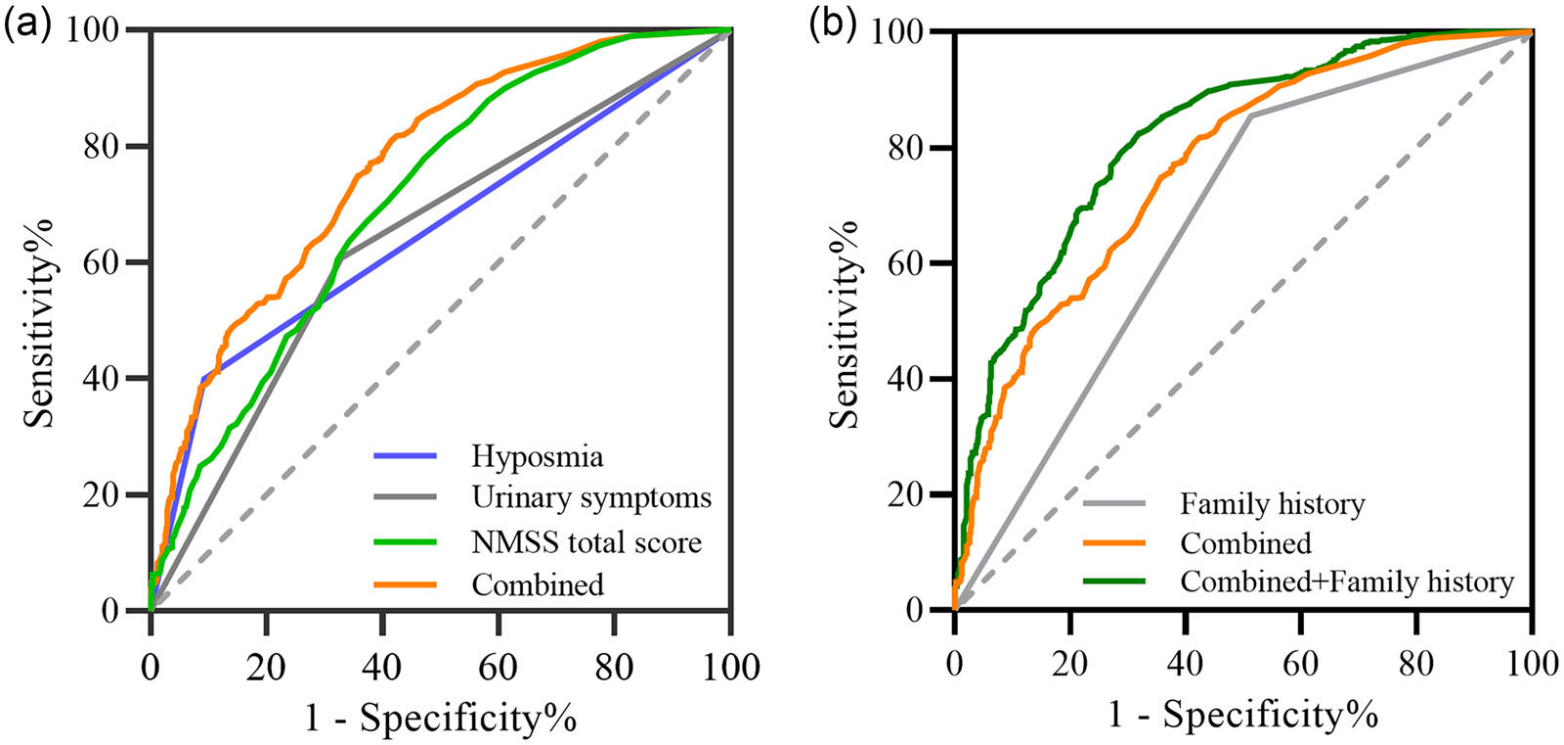
Differentiating ROC curves for ET and PD‐TD. (A) NMS distinguishes between ET and PD‐TD; (B) NMS and family history differentiate ET from PD‐TD.

Univariate and multivariate logistic regression analyses were conducted on the NMS of ET and PD‐TD (Table S2). It was found that the NMSS total score, urinary symptoms, and hyposmia are independent risk factors for distinguishing ET from PD‐TD. Based on the ROC analysis, the AUC for the NMSS level difference was 0.706 (95% CI: 0.677–0.736), the AUC for the urinary symptom difference was 0.639 (95% CI: 0.607–0.672), and the AUC for the hyposmia difference was 0.654 (95% CI: 0.622–0.686). A higher AUC of 0.766 (95% CI: 0.739–0.793) was achieved when combining NMSS total score, urinary symptoms, and hyposmia, exhibiting a sensitivity of 80.8% and a specificity of 58.6% (Figure 4A). Incorporating family history into the model further improved the AUC to 0.819 (95% CI: 0.795–0.843) (Figure 4B), with a sensitivity of 82.4% and a specificity of 68.2%, thus enhancing the differentiation between ET and PD‐TD.

## 4 Discussion

ET and PD‐TD often exhibit overlap in motor symptoms, and the characteristics of NMS in these diseases have not been fully elucidated. In our study, we expanded the sample size to compare the NMS differences among ET, PD‐TD, and a control group. The results indicated that patients with PD‐TD exhibited a greater number and severity of NMS compared to those with ET. These diseases exhibit significant differences in NMS, particularly in NMSS scores, urinary symptoms, and hyposmia. Accurately identifying these distinct NMS is crucial for assessing and developing management strategies and diagnostic procedures for ET and PD‐TD.

We conducted a retrospective study involving patients diagnosed with PD‐TD and ET, revealing that PD‐TD patients tend to have a later AAO and a shorter disease duration, aligning with previous research (Kwon et al. 2016; Shalash et al. 2021). Additionally, our study corroborated that ET is associated with a more prominent positive family history (Welton et al. 2021). We observed certain differences in motor symptoms between PD‐TD and ET patients; notably, PD‐TD patients exhibited a significantly higher asymmetry in limb tremors (71.86% vs. 23.97%), consistent with prior studies (Kwon et al. 2016; Louis et al. 1998; Minen and Louis 2008). Head tremors were predominantly observed in ET patients over PD‐TD, with our study finding head tremors in only 15 out of 558 PD‐TD patients. In contrast, head tremors are relatively rare in PD (Jankovic 2008). Furthermore, our findings indicated that lower limb tremors were more prevalent in PD‐TD than in ET patients.

Despite these distinctions, some patients still exhibit symptom overlap. Head tremors play a significant role in differentiating the two diseases, yet approximately 70% of ET patients do not experience head tremors. Therefore, we compared NMS between ET and PD‐TD to provide more evidence for diagnosis and management.

This study analyzed and found significant differences between ET and PD‐TD in terms of NMSS total scores, urinary symptoms, and hyposmia. The combination of NMSS total score, urinary symptoms, and hyposmia partially reflected the differences between ET and PD‐TD, with an AUC of 0.766 (95% CI: 0.739–0.793), a sensitivity of 80.8%, and a specificity of 58.6%. Despite the lower specificity, which may lead to some false positives, the high sensitivity is important for identifying more PD‐TD patients, especially in the early stages where clinical diagnosis may be more challenging. Further analysis incorporating family history into the model improved the combined AUC to 0.819 (95% CI: 0.795–0.843), with a sensitivity of 82.4% and specificity of 68.2%. This enhanced model strengthens the evidence for distinguishing between ET and PD‐TD, and the improved specificity reduces false positives without compromising sensitivity.

The NMSS score represents the severity of NMS. Our comparison of NMS severity across three groups showed that PD‐TD patients experience more severe NMS than both ET and control groups, similar to findings by Shalash et al. (2021). However, research by Lee et al. (2015) found ET to share similarly severe NMS with PD (ET: 25.500 ± 2.346; PD: 27.960 ± 3.267), whereas Kwon et al. (2016) reported no significant difference in NMS severity between the two. In the Chinese population, the average NMS score for ET is around 12 (H.‐Y. Huang et al. 2020; Sun et al. 2023), while PD patients have an average score of 36.06 (Z. Zhou et al. 2021), indicating more severe NMS in PD patients. The severity of NMS could aid in distinguishing PD‐TD from ET patients. The NMS scores for ET were similar to those of the control group, possibly due to the older age of control participants (60.93 ± 19.79 years). Yet, ET patients generally had a higher frequency of NMS than the control group, especially regarding mood issues, supporting that NMS is part of the ET phenotype (Louis 2016). It is noteworthy that research by Giorelli et al. (2014) suggests ET patients may experience spontaneous remission of NMS.

PD‐TD patients showed a higher frequency of NMS than ET patients. Research by Shalash et al. (2021) found PD‐TD more frequently exhibited mood, memory/attention, gastrointestinal tract, urinary, miscellaneous, and depression issues than ET. The study of Kwon et al. (2016) highlighted that olfactory decline, RBD‐like symptoms, urinary frequency, and memory impairment were more common in PD‐TD than ET. Our study found PD‐TD more frequently associated with mood, urinary symptoms, fatigue, constipation, hyposmia, and hyperhidrosis. Although these studies revealed frequency differences in NMS between PD‐TD and ET, overall, PD‐TD patients had a higher risk of NMS. No difference was found in insomnia between ET and PD‐TD (Kwon et al. 2016); in fact, insomnia is common in ET, with studies indicating 12.5%–56.6% of ET patients experiencing insomnia (Jiménez‐Jiménez et al. 2020). In our study, 41.95% of ET patients reported insomnia. Consolidating the conclusions above, it is evident that the studies mentioned highlight the differences in NMS between ET and PD‐TD, particularly in terms of hyposmia and urinary symptoms. These findings are consistent with our study, indicating that urinary symptoms and hyposmia exhibit significant differences between ET and PD‐TD patients.

Notably, the frequency of hyposmia decline in PD‐TD is 7.7 times that of ET, suggesting a greater advantage in differentiating PD‐TD from ET, with several studies supporting this outcome (Busenbark et al. 1992; Elhassanien et al. 2021; Shah et al. 2008; Wolz et al. 2014). Shah et al. (2008), using the University of Pennsylvania Smell Identification Test (UPSIT) and olfactory event‐related potential (OERP), assessed olfactory function in 59 ET patients and 64 PD‐TD patients, finding ET patients scored higher on UPSIT and had better OERP olfactory detection than PD‐TD patients. Elhassanien et al. (2021) conducted the *n*‐butanol threshold sniffing test (SST) and olfactory bulb volume measurements (OBV) on 36 ET patients and 22 PD‐TD patients, discovering a significant decline in SST total score (TDI) among PD‐TD patients compared to ET, suggesting TDI can differentiate PD‐TD from ET patients with a sensitivity and specificity of 94% and 91%, respectively. Thus, olfactory testing may play a role in differentiating PD‐TD from ET.

Although our study provides valuable insights, it also has certain limitations. For instance, this was a retrospective study, which may be subject to incomplete data or biases. Additionally, there were significant differences among the three groups included in the study. Most importantly, our differentiation between ET and PD‐TD was based on the NMSS, limiting our assessment of other NMS, such as anxiety, depression, RBD, and restless legs syndrome (RLS), which were not evaluated. Future studies should consider employing a longitudinal design and expanding the sample size to allow for a comprehensive assessment of NMS, further exploring the role of NMS in differentiating and managing ET and PD‐TD.

In summary, our research highlights the importance of considering NMS in the differential diagnosis of ET and PD‐TD. Identifying NMS not only assists clinicians in making more accurate diagnoses, offering more personalized treatment options for patients, but also signifies potential pathophysiological and neurodegenerative differences, providing a basis for further mechanistic exploration. As our understanding of these diseases deepens, future diagnostic criteria and treatment guidelines should more comprehensively consider the impact of NMS.

## Author Contributions

**Mingqiang Li**: investigation, formal analysis, writing–original draft. **Runcheng He**: investigation, formal analysis, writing–original draft. **Xun Zhou**: investigation, formal analysis. **Yuzheng Wang**: investigation, formal analysis. **Qiying Sun**: investigation, formal analysis, writing–review and editing. **Chunyu Wang**: investigation. **Sheng Zeng**: investigation. **Lifang Lei**: investigation. **Heng Wu**: investigation. **Shanqing Yi**: investigation. **Jun Wen**: investigation. **Qian Xu**: investigation. **Jifeng Guo**: investigation. **Beisha Tang**: investigation, writing–review and editing, funding acquisition.

## Conflicts of Interest

The authors declare no conflicts of interest.

### Peer Review

The peer review history for this article is available at https://publons.com/publon/10.1002/brb3.70288.

## Supporting information



Supporting Information.

## Data Availability

The data for this study may be shared upon reasonable request to the corresponding author.
