# Supplementary material for: Nonmotor Symptoms Differ Between Essential Tremor and Tremor‐Dominant Parkinson's Disease
Source: Brain Behav. 2025 Feb 9;15(2):e70288. doi: 10.1002/brb3.70288 (PMC11808177; doi:10.1002/brb3.70288)
Supplement: Supplementary file 1 — Supporting Information. [file BRB3-15-e70288-s001.docx]

**Non-motor symptoms differ between essential tremor and tremor-dominant Parkinson's disease**

**Supplementary Material**

Table S1 Number of NMS in ET, PD-TD and control

| Number of NMS | Control (514) | PD-TD (558) | ET (584) |
| --- | --- | --- | --- |
| NMS (%) | 54.86 | 94.44 | 71.23 |
| 0 | 232 | 31 | 168 |
| 1 | 142 | 95 | 126 |
| 2 | 87 | 93 | 94 |
| 3 | 34 | 131 | 86 |
| 4 | 15 | 93 | 61 |
| 5 | 4 | 66 | 36 |
| 6 | 0 | 39 | 12 |
| 7 | 0 | 10 | 1 |

Table S2 Univariate and multivariate logistic regression analysis of NMS factors differentiating ET and PD-TD

| NMS | Univariate | | Multivariate | |
| --- | --- | --- | --- | --- |
|  | *P*-value | OR (95% CI) | *P*-value | OR (95% CI) |
| MMSE | 0.003 | 0.95 (0.91-0.98) | 0.845 | 1.00 (0.95-1.03) |
| NMSS total score | 0.000 | 1.04 (1.03-1.05) | 0.050 | 1.01 (1.00-1.02) |
| Mood | 0.000 | 1.70 (1.34-2.15) | 0.634 | 0.93 (0.69-1.26) |
| Urinary symptoms | 0.000 | 3.16 (2.48-4.03) | 0.000 | 2.19 (1.64-2.93) |
| Fatigue | 0.000 | 2.15 (1.69-2.74) | 0.194 | 1.22 (0.90-1.65) |
| Insomnia | 0.467 | 1.09 (0.86-1.38) | - | - |
| Constipation | 0.000 | 2.30 (1.77-2.99) | 0.123 | 1.28 (0.94-1.74) |
| Hyposmia | 0.000 | 6.53 (4.71-9.06) | 0.000 | 4.97 (3.50-7.05) |
| Hyperhidrosis | 0.011 | 1.47 (1.09-1.97) | 0.727 | 0.94 (0.67-1.32) |
